# Living with cystic fibrosis during the COVID-19 pandemic: An interpretive description of healthcare access from patients with cystic fibrosis and their providers in Alberta, Canada

**DOI:** 10.1371/journal.pone.0322911

**Published:** 2025-05-02

**Authors:** Katelyn Brehon, Pam Hung, Maxi Miciak, Winnie M. Leung, Kadija Perreault, Douglas P. Gross, Jason Weatherald, Paul E. Ronksley, Michael K. Stickland, Grace Y. Lam

**Affiliations:** 1 Faculty of Rehabilitation Medicine, University of Alberta, Edmonton, Canada; 2 Division of Pulmonary Medicine, Department of Medicine, University of Alberta and Alberta Health Services, Edmonton, Alberta, Canada; 3 Alberta Respiratory Centre, University of Alberta, Edmonton, Alberta, Canada; 4 Département de readaptation, Université Laval, Laval, Canada; 5 Department of Physical Therapy, University of Alberta, Edmonton, Canada; 6 Department of Community Health Sciences, Cumming School of Medicine, University of Calgary, Calgary, Alberta, Canada; 7 O’Brien Institute for Public Health, University of Calgary, Calgary, Alberta, Canada; University of Minnesota, UNITED STATES OF AMERICA

## Abstract

**Background:**

The current study aimed to explore patient and provider perspectives of the impact of the pandemic on cystic fibrosis healthcare access and service delivery.

**Methods:**

We used Interpretive Description, a qualitative approach with the end-goal of informing decisions and actions in clinical practice by generating findings that are clinically meaningful and useful. Levesque *et al.’s “*Conceptual framework of access to health care” informed the development of our interview guides. Interviews were conducted via telephone or Zoom and confidentially transcribed verbatim. Data generation and analysis occurred concurrently to allow for iterative refinements of the interview guides. Analysis was informed by Braun and Clarke’s six phases of reflexive thematic analysis. Strategies to enhance rigour and trustworthiness of the findings were utilized.

**Results:**

We completed 13 interviews: 8 with patients and 5 with providers. Three key themes were generated: (a) Tensions due to infection prevention at micro- meso-, and macro- levels; (b) Modifying aspects of person-focused care can bolster perceived quality of clinical encounters; and (c) Accessibility of appropriate healthcare services could improve efficiency of service delivery. Infection prevention at the individual level was not found to be burdensome. Society’s compliance with public health measures, or lack thereof, impacted the level of stigma and anxiety experienced by patients with cystic fibrosis. A changed model of care reliant on patient self-report instead of clinician-led testing and in-person assessment due to the transition to virtual care was associated with mixed perceptions since patients with cystic fibrosis were comfortable making care decisions but many participants (patient and provider) felt challenged by the lack of objective data for decision-making. It was essential for patients with cystic fibrosis to feel known, heard, and seen by their providers in order to feel the care was effective. Finally, critical insights around the need for a balance of in-person and virtual care as well as the need for mental health supports were offered.

**Conclusions:**

The learnings from this study could be translated into practical strategies for improving cystic fibrosis care during the pandemic and beyond. We recommend: (1) a hybrid approach to care moving forward, (2) each patient having a lead physician with others filling in as necessary when scheduling demands, and (3) a reallocation of resources to fund a mental health practitioner position.

## 1. Introduction

The COVID-19 pandemic impacted the health of patients globally. For people with chronic pulmonary conditions, the pandemic presented new challenges and barriers to accessing healthcare. Some pulmonary diseases, like tuberculosis, saw lower incidences but greater mortality due to missed diagnoses [1–3]. Other conditions, like chronic obstructive pulmonary disease, reported decreased emergency department visits, hospitalizations, and mortality [4–6].

Preliminary data from Alberta showed that emergency department visits and hospitalizations for the most common lung diseases, chronic obstructive pulmonary disease and asthma, decreased significantly during the early months of the COVID-19 pandemic [6]. However, these data provide an incomplete picture of overall access to healthcare, health resource utilization, and outcomes, with important knowledge gaps remaining. Importantly, there is little data on healthcare use and outcomes for patients with rare lung diseases despite the fact that such individuals have complex care needs and high morbidity. These individuals require specialized care that in Alberta, is usually centralized in major urban centres and at the time of the current study, was provided under one provincial health authority. This existing model of centralized care under one health authority may have disproportionately impacted some patients but further information from this context is needed.

Cystic fibrosis (CF) is an autosomal recessive hereditary condition primarily affecting the lung, which is the most common cause of morbidity and mortality in patients with CF (pwCF). CF can also manifest in the sinuses, gastrointestinal tract, pancreas, reproductive tract and others [7]. Studies of healthcare utilization by pwCF during the pandemic showed that care predominantly shifted to telehealth delivery with less healthcare resources accessed in-person [8–11], though the impact of this transition on CF-relevant health outcomes is unclear. Some studies highlighted improvements in lung function and mental health as well as reduced number of pulmonary exacerbations, reflective of possible improved control of the disease [12–14]. Other studies noted a detrimental delay to care as a result of the pandemic [15]. These differences could potentially be due to variation across systems or modes of service delivery, although little is known.

Qualitative research investigating how the rapid changes necessitated by the pandemic may have created unintended barriers to accessing healthcare and subsequently impacted the health of adult pwCF is mixed and limited. A multiple methods study (n = 15 patient interviews) aimed at understanding the impact of the pandemic and associated public health measures on the management, health, and behaviours of pwCF during the first wave of COVID-19 in France found that pwCF had appointments cancelled and postponed with some moved to telehealth delivery [16]. In contrast, a qualitative study of partners of women with CF (n = 20) and their healthcare providers (n = 20) exploring perceptions of telehealth into routine CF care found that newly adopted telehealth delivery increased connection between the healthcare team and the family of pwCF, improved efficiency of healthcare delivery, and improved interdisciplinary connection [17]. To our knowledge, perceptions of pwCF and their healthcare providers on access to care and service delivery during the pandemic have not been explored in our context. As such, the objective of this study was to explore patient and provider perspectives on the impact of the pandemic on healthcare access and service delivery experienced by pwCF as well as patient health with the goal of identifying implementable strategies for health system improvements post-pandemic.

## 2. Methods

This study was conducted in Alberta, Canada. Ethics approval was obtained from the University of Alberta’s Health Research Ethics Board (Pro00118367). All participants provided informed verbal consent. Verbal consent was documented on the study consent form by the study coordinator and then emailed to each participant for their records following the interview.

### 2.1 Researchers’ positionality

The interdisciplinary research team consisted of clinicians and researchers with diverse levels of experience and various professional backgrounds in pulmonology, public health, rehabilitation, health services, and qualitative and quantitative methodologies. Some members were “insiders” providing pulmonary care during the pandemic whereas others were “outsiders” bringing diverse research and clinical experiences outside of pulmonary care to the study [18]. The first author, who was the interviewer and primary analyst, has a background in public health and qualitative inquiry, but not as a clinician. Therefore, the various perspectives and balance of “insiders” and “outsiders” included during data analysis offered diverse and important insight into how the data were interpreted and the clinical relevance of the findings [18].

### 2.2 Methodological approach

We used Interpretive Description (ID), a qualitative approach with the end-goal of informing decisions and actions in clinical practice by generating findings that are clinically meaningful [18–20]. ID aligns with the constructivist paradigm, highlighting participants’ experiences and knowledge to provide in-depth characterization of a phenomenon [20]. Findings are co-constructed, a result of the researchers’ inductive interpretations of participant experiences within the context and knowledge of the applied discipline (e.g., medicine, rehabilitation) [18,20]. ID is a flexible approach that enables thorough exploration of complex and practical questions [20].

### 2.3 Conceptual framework

Health systems, and individuals’ experiences of navigating them, are complex. We used Levesque et al’s ‘Conceptual framework of access to health care’ to help us consider concepts relevant to systems and how interactions between patients’ abilities to perceive, seek, reach, pay, and engage in healthcare services and the health system influence access [21,22]. Levesque’s framework has been used in health services [21,23], rehabilitation [24,25], and global health [26] research. We used the framework to inform the development of semi-structured interview guides.

### 2.4 Study population and recruitment

In Canada, CF care can be separate for pediatric and adult patients or combined in one clinic for all age ranges and is centralized in designated provincial clinics across the country. Rural communities are served by their nearest CF clinic. The centralized clinics are multidisciplinary in nature, led by the physician, and can include nurse practitioners, registered nurses, physiotherapy, social work, dietetics, and pharmacy. PwCF typically have clinic appointments three to four times per year. CF specialty care clinics in Alberta operate in a similar multidisciplinary manner and are centralized in two urban centres: Edmonton and Calgary. While the goal was to interview patients and providers from both urban centres, participants were only recruited from one adult clinic in one urban centre due to recruitment challenges.

We aimed to recruit 8–10 pwCF and 6–8 provider participants. This sample size was perceived as feasible within the study timeline and is sufficient for qualitative analysis in ID studies [18]. There are also limited providers working in the clinics of interest which limited the number of participants who could be recruited. Participants were recruited between October 17, 2022 and December 15, 2022. All participants were adult residents of Alberta, were willing to participate in research, and were able to provide informed consent. PwCF also needed to be diagnosed with CF prior to the beginning of the COVID-19 pandemic (March 2020 in Canada). We aimed to recruit adult pwCF who had received care in the adult CF clinic of interest prior to the pandemic. There was no minimum number of pre-pandemic appointments required to be included in the study. Similarly, provider participants needed to be working in a CF clinic prior to the beginning of the pandemic. Providers were not required to have a specific minimum number of years of experience. There were no specific inclusion criteria regarding professional designation and we attempted to gain insight from a variety of professionals including physicians, nurse practitioners, and allied health team members.

Purposive sampling towards maximum variation directed patient recruitment. The three primary diversification criteria included age, gender, and geographical location (rural or urban). A diverse sample of pwCF who met the inclusion criteria were identified and approached by healthcare providers involved in their care (not members of the research team) who introduced the study and confirmed consent to share contact information with the research team. The study coordinator (KB) followed up with each eligible and interested pwCF to provide further information, gain informed consent, and schedule an interview.

All providers working in the included clinic were sent an email by a fellow clinician who is also a member of the research team. The email introduced the study and provided contact information for the study coordinator. Providers willing to participate contacted the study coordinator who provided additional information about the study, gained informed consent, and scheduled an interview. Only one follow-up email was sent to aid in recruitment while also minimizing pressure to participate.

### 2.5 Data generation

Data generation occurred between October and December 2022. At this time, Alberta was experiencing its seventh COVID-19 wave after several rounds of tightening and relaxing public health protocols. General advice was to mask, isolate when sick for five days or until asymptomatic (whichever was longer), and social distance whenever possible. There was no national lockdown and no national guidance for pwCF.

One-on-one semi-structured interviews were completed by one interviewer (KB) via videoconference (Zoom) or phone. Separate interview guides composed of open-ended questions (see Appendix 1) addressing need for care, access to care, and quality and effectiveness of care were developed for pwCF and providers. Probing questions explored topics in greater depth and detail, as needed. Data generation and analysis occurred concurrently, enabling iterative refinements to the interview guides [18].

Participants were asked open-ended questions to facilitate self-report of gender and cultural or ethnic background as well as the first three digits of their postal code (patients) or years of professional experience (providers). These questions were open-ended to ensure participants used terminology meaningful to them. These data were collected with the intention of understanding and describing the sample rather than for making comparisons during analysis. Interviews were recorded, transcribed verbatim, reviewed for accuracy, and imported into NVivo for analysis [27].

### 2.6 Data analysis

The analysis was guided by Braun and Clarke’s six phases of reflexive thematic analysis [28–31]. The primary study analyst (KB), who also conducted the interviews, began by cleaning and re-reading all transcripts to further familiarize with the data and generate initial analytic thoughts. Next, initial codes were developed inductively by KB for each transcript. Codes were considered in relation to one another and expanded or collapsed based on patterns of meaning.

**Fig 1:**
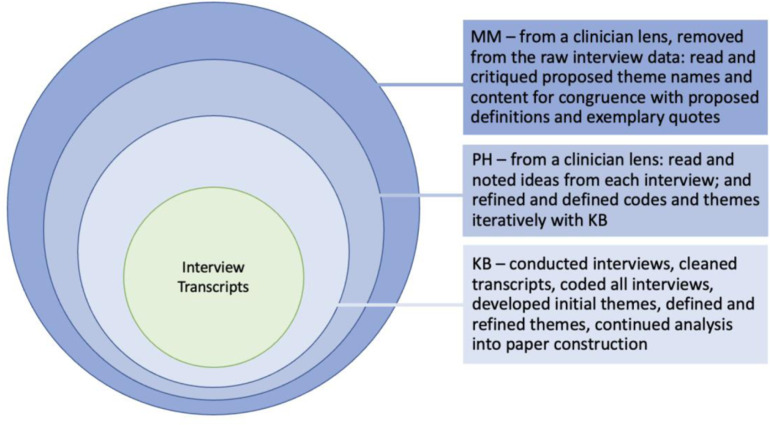
Layers of analytic critique used in relational analysis process.

To enhance rigour, a relational approach to analytic critique (Fig 1) was used to generate dialogue between KB, PH, and MM, aiding in the co-construction of findings. After KB completed the initial construction of codes and preliminary themes, two researchers (PH and MM), who are both rehabilitation providers with experience in qualitative methodologies including ID were engaged to further push the analysis in terms of analytic logic and interpretive authority [18]. PH was involved at the raw data level, becoming familiar with the data by reading each transcript and noting initial ideas from each interview. KB and PH met to discuss important ideas, including clinical relevance of information, and went through iterations of potential themes and subthemes via discussion, critique, and reflection. Themes and subthemes were then named, defined, and exemplary quotes retrieved from the transcripts and shared with MM, who had not read the transcripts. MM provided critical methodological and clinical perspectives on: (1) internal congruence within themes to ensure information contained within themes and subthemes was unique; and (2) external congruence among themes to ensure themes and subthemes did not overlap. Themes and subthemes went through further discussion and critique between KB, PH, and MM until finalized.

To further enhance credibility of the analysis, KB contacted each participant to ask whether they would provide insights on the resonance of proposed themes and subthemes [32]. Two provider participants agreed whereas no pwCF agreed. The two provider participants were emailed the proposed themes, subthemes (including definitions), and exemplary quotes and were asked to respond to the following questions (Appendix 2): (1) Do these findings resonate with your experience of providing CF care during the pandemic; (2) If an idea does not resonate, why not and what could we change to make it resonate more; and (3) Is there anything key to your experience that you think is missing? Any questions or concerns were noted and reflected upon. Both providers shared that findings resonated with their experiences with one highlighting further queries about the data that were outside of the scope of the study, which therefore did not result in changes. Analysis continued while the final report was drafted and included integration of provider participant feedback.

### 2.7 Rigour

Strategies to enhance epistemological integrity, representative credibility, analytic logic, and interpretive authority were utilized [18] to enhance trustworthiness. ID supports the integration of clinical ways of knowing and context in data generation and analysis, which was important for co-constructing findings that were clinically important and useful. As such, we ensured epistemological integrity of the study by choosing ID to address our exploratory objective [18–20]. Representative credibility was enhanced by interviewing both patients and providers to ensure both groups contributed to the co-construction of knowledge. Analytic logic was enhanced through an audit trail of decisions of accountability and thick description to prioritize verbatim participant accounts. Interpretive authority was enhanced through constant reflection about potential biases or experiences that could impact interpretation during analysis.

## 3. Results

### 3.1 Participant characteristics

We completed 13 interviews: 8 with patients and 5 with providers. Table 1 (patient) and Table 2 (provider) outline participant characteristics. Most patient (62.5%) and provider (100.0%) participants self-identified as female. Most pwCF lived in an urban centre and had a median (interquartile range) age of 32.0 years (26.3–50.5). Provider participants ranged in years of experience from 2 to 16 years with a mean (SD) of 11.0 (5.7) years (the individual with 2 years of experience had only been *on staff* for 2 years but had experience with the population during training prior to the pandemic). On average, interviews with providers tended to last longer (46.5 ± 7.8 minutes) than with patients (38.9 ± 17.4 minutes).

**Table 1:** Patient participant characteristics.

Variable	N (%) or Median (Interquartile Range (IQR))
Gender^a^	
Male	3 (37.5%)
Female	5 (62.5%)
Median age (years) (IQR)	32.0 (26.3-50.5)
Rural/Urban	
Rural	3 (37.5%)
Urban	5 (62.5%)
Ethnicity^b^	
Caucasian	6 (75.0%)
Indigenous	1 (12.5%)
Lebanese	1 (12.5%)
Chose not to disclose	1 (12.5%)

^a^All participants were asked their gender as an open-ended question in conversation with the interviewer. All responded using sex-based categories of male/female. We are reporting terms that participants used.

^b^All participants were asked to identify their cultural or ethnic background in an open-ended question in conversation with the interviewer. We are reporting terms that the participants used. One individual identified with more than one ethnic background which is why the total does not add up to 100.0%.

**Table 2:** Provider participant characteristics.

Variable	N (%), Median (IQR), or Mean (standard deviation (SD))
Gender^a^	
Male	0 (0.0%)
Female	5 (100%)
Median age (years) (IQR)	45.0 (39.5-56.5)
Mean experience (years) (SD)	11.0 (5.7)
Ethnicity^b^	
Asian	2 (40.0%)
Caucasian	1 (20.0%)
English	1 (20.0%)
Hungarian	1 (20.0%)
Iranian	1 (20.0%)
Persian	1 (20.0%)

^a^All participants were asked their gender as an open-ended question in conversation with the interviewer. All responded using sex-based categories of male/female. We are reporting terms that participants used.

^b^All participants were asked to identify their cultural or ethnic background in an open-ended question in conversation with the interviewer. We are reporting terms that the participants used. Two individuals identified with more than one ethnic background which is why the total does not add up to 100%.

### 3.2 Impact of the pandemic on healthcare access, service delivery, and health of pwCF

**Fig 2:**
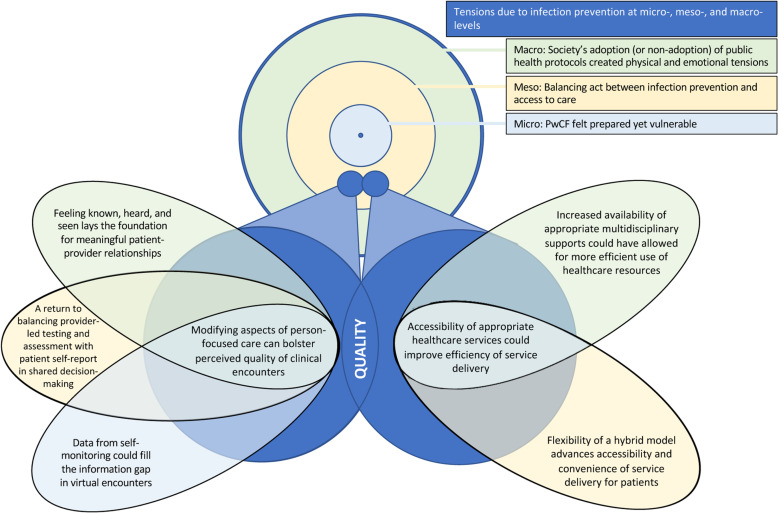
Thematic framework depicting the relationships between themes and subthemes. pwCF: Patients with Cystic Fibrosis.

Three key themes were generated: (a) Tensions due to pandemic-related infection prevention at micro- meso-, and macro- levels; (b) Modifying aspects of person-focused care can bolster perceived quality of clinical encounters; and (c) Accessibility of appropriate healthcare services could improve efficiency of service delivery. The relationships between themes and sub-themes are depicted in Fig 2. Additional exemplary quotes can be found in Appendix 3.

#### 3.2.1 Theme A: Tensions due to pandemic-related infection prevention at micro-, meso-, and macro- levels.

The first theme expresses the tensions experienced by pwCF due to pandemic-related infection prevention at the individual, patient-level; the broader circle of support level (i.e., family, friends, and healthcare providers); and at the societal level.

*Micro: PwCF felt prepared yet vulnerable.* On a micro-level, pwCF believed that their lifelong infection prevention efforts worked to their advantage during the pandemic because “*the current procedures [were] … pretty much what [the clinic asked us to do] pre-COVID … They kind of [were] treating us all like little COVID patients, pre-COVID*” (patient 4, female). This lifetime of infection prevention meant that pwCF seemed to feel a sense of readiness when COVID-19 became a global threat: *“for me [COVID] didn’t feel like anything new”* (patient 8, male).

Provider participants also perceived patient preparedness: “*in the context of the pandemic [CF patients have] been training for this their whole life. They understand the importance of … staying healthy and washing their hands and mitigating risk*” (provider 5, female). One provider participant highlighted how the rest of the world had to adjust to protocols of which pwCF were already well versed: *“for our patients it’s kind of like they were living COVID protocol … their entire lives. And then it’s just the rest of the world caught on”* (provider 1, female).

There was, however, increased individual-level anxiety, *“I was … really anxious about catching COVID at some point, especially pre-vaccine”* (patient 4, female), and vigilance, *“I’m very careful about where I’m going, when I’m going, … trying to avoid lots of people, wash my hands. I always have a mask with me”* (patient 3, female), associated with feelings of vulnerability due to the pandemic. Provider participants recognized how patient anxiety about COVID-19 left pwCF feeling vulnerable, sometimes leading them to have increased interest in accessing care:

*“if they did get sick then they’re more afraid of COVID so they want to know what the clinic thinks, so I think [we] got more phone calls with sick patients thinking, oh my God, … I need to know from the clinic what they want me to do.”* (provider 2, female)

In comparison, providers perceived that patient anxiety and feelings of vulnerability also prevented some from accessing care even if providers thought it was the most appropriate course of action:

*“we did have some people where they were potentially ill but afraid to come in. … we would have … wanted them to come in [and] there was availability in the facility to bring them in … but unfortunately the patient was just not comfortable.”* (provider 4, female)

This tension between feeling prepared and experiencing anxiety associated with increased vulnerability at the individual-level highlights that even though pwCF were used to preventing infections and felt comfortable taking precautions, discomfort with the unknowns of COVID-19 sometimes impacted their willingness to access care.

*Meso: Balancing act between infection prevention and access to care.* PwCF recognized how providers seemed to weigh the pros and cons of in-person appointments during the pandemic as there was little understanding of how pwCF would fare if they caught COVID-19:

*“During the pandemic … [providers] definitely had their hands full, like it’s people who were immunocompromised that they’re dealing with constantly … so it’s very taxing for them as well … They have so many basically sick patients that need to come in, but again it’s a pandemic, so it’s they’re playing a game of do we want these patients in right now”* (patient 8, male)

Similarly, provider participants discussed decisions they wrestled with to balance standardized CF care with the need for infection prevention:

*“I don’t know if I was able to provide the best care because I haven’t been able to see them in-person … But in terms of harm reduction, mitigating exposure and things like that, that might have been safer for the patients because they didn’t have to come here and get exposed or risk exposing other people to possible early COVID… whether bringing them in was a good idea [or not] … was a big consideration … it was a bit of a balance”* (provider 1, female)

Ultimately, pwCF seemed to appreciate the balance struck by providers between standardized care and infection prevention. Some pwCF perceived the quality of care during the pandemic as better than what was provided before the pandemic due to increased vigilance with infection prevention practices: *“I think the care during the pandemic, … the quality was probably a little bit better because maybe they were on high alert, just because everybody would see us as more on the … riskier zone”* (patient 6, female).

Outside the clinical setting, pwCF spoke about increased vigilance with infection prevention amongst family members and friends, though some felt that COVID-19 public health measures (i.e., masking indoors, quarantining, social distancing) “*w[ere]n’t really anything new*” (patient 6, female). Increased worry and associated vigilance by loved ones sometimes created frustration amongst pwCF. PwCF sometimes felt more limited than they had been pre-pandemic due to their loved ones’ newfound worries, which created an unintended barrier to accessing healthcare for pwCF:

*“…the tough part was just dealing with family members … [who] got very panicked about it. And obviously they don’t want me to get sick so … they didn’t want me going up to places or going anywhere really to even pick up my meds or anything like that … It’s frustrating… So that was the main … barrier that I had.”* (patient 8, male)

*Macro: Society’s adoption (or non-adoption) of public health protocols created physical and emotional tensions.* PwCF were physically and emotionally impacted by society’s uptake (or lack thereof) of public health protocols. Physically, pwCF described that before the pandemic, it would be common to get sick during the cold and flu season; however, both patient and provider participants revealed that the widespread public health measures instituted during the pandemic resulted in pwCF feeling the healthiest they had ever been: “wearing the mask really did make a difference … like I said, I felt my healthiest I’ve ever been” (patient 7, female); *“we’ve certainly had patients … tell us … ‘the last two years I felt the best I’ve ever felt’ … because [of] the public health measures”* (provider 1, female).

Nevertheless, some pwCF contracted COVID-19 outside of the clinical setting during the pandemic. Patient and provider participants spoke about how COVID-19 outcomes were better than expected:

“*… there was an expectation setting … because we were worried that our CF patients would be more vulnerable to COVID and that they would be worse if they got COVID but we actually didn’t see that. … [but] we were all prepared for the worst*” (provider 1, female)

*“my lungs were completely fine [with COVID], which I was very surprised ‘cause everyone kept saying they couldn’t breathe, … but … my lungs … held up and it was just my joints, I could barely walk kind of thing. But … like it wasn’t as bad as what … I was preparing myself for, I guess?”* (patient 7, female)

Emotionally, public health protocols affected pwCF in a variety of ways. When encountering members of the public practicing public health measures, pwCF sometimes experienced increased empathy. Experiencing increased empathy seemed to depend on encountering individuals who knew about their CF diagnosis:

*“People … maybe understand a bit more how difficult it might be to live with CF. Because maybe people are thinking more about the difficulty of having a chronic lung condition because [COVID] is based on the lungs … But … [that’s] people who I got to talk to and say like, hey, this is what I go through, I have CF.”* (patient 2, male)

PwCF also spoke about experiencing increased stigma around having a chronic cough. This stigmatization seemed to be associated with encountering individuals who were unaware of their CF diagnosis and attributed their symptoms to COVID-19:

*“I was standing in line [at the grocery store] and … there [was] this older gentleman behind me, and I had a coughing fit, because my lungs are mucousy … but he doesn’t know that. … I had my mask on and everything’s good … [but] he turned and looked at me and he actually left the line and went to a different … line that was … 10 times longer, because he just didn’t want to be around me.”* (patient 8, male)

Some pwCF spoke about feeling anxious if they were in a setting where others might be dishonest about their symptoms or if they encountered individuals who were not following public health measures, which sometimes led to social isolation:

*“being in proximity to a maskless person can really raise my anxieties around me going out in public … I was aware that a lot of people were refusing to comply. … [J]ust being concerned that I might cross paths with somebody who was not compliant with the guidelines stopped me from pursuing activities outside of my house a lot of the time.”* (patient 2, male)

In sum, the adoption of public health measures could have enabling or disabling impacts, highlighting how society’s responses could affect pwCF’s willingness to engage publicly or seek appropriate health services.

#### 3.2.2 Theme B: Modifying aspects of person-focused care can bolster perceived quality of clinical encounters.

In theme (b), participants spoke about pwCF feeling *known, heard*, and *seen*; a return to balancing patient self-reported accounts and physician-led care in shared decision-making; and using self-monitoring to fill information gaps during virtual appointments.

*Feeling known, heard, and seen lays the foundation for meaningful patient-provider relationships*. The CF clinic included in the current study, as with many clinics in Canada, operates under a physician shared care model (patients see whatever physician is working rather than one specific physician). PwCF expressed some disfavour with the lack of consistency of their main provider, which was a feeling felt prior to the pandemic. PwCF discussed not feeling *known* by providers if they were frequently seen by different practitioners, which contributed to decreased perceptions of quality in the patient-provider relationship:

*“[In] the CF clinic, … I think [there]’s four doctors. … I went for my yearly clinic, and … the doctor … called me by the wrong name. I don’t think [they] had the right chart up on the computer. … so from that point forward, I always see the same doctor, and I’ve made it quite clear, unless it’s an emergency, I only want to see the one doctor … once I switched to a single doctor … my quality of care has been – I have nothing but good things to say about it.”* (patient 3, female)

Ability for pwCF to easily call and talk to providers at the clinic contributed to them feeling *heard* by their team, thus bolstering perceived quality in the patient-provider relationship. Patient and provider participants discussed how pwCF knew that they could contact the clinic at any time regarding any queries or concerns both before and during the pandemic:

*“… our patients know that they can just call the clinic. … there’s somebody there all the time that they know and that they can talk to. So that’s kind of the unique thing about [CF], whereas other lung conditions … wouldn’t have that capability”* (provider 1, female)

The ability of pwCF to easily communicate with their care team (i.e., physician, nurse, physiotherapist, social worker, dietician) was paramount during the pandemic when there were periods of increased uncertainty and a lack of clarity around factors associated with COVID-19 management: *“I was able to always email if I had any problems or questions [during the pandemic] … they definitely always answer[ed] my questions or concerns if I had any, especially when I had COVID”* (patient 7, female).

After experiencing the delivery of mainly virtual care during the pandemic, patient and provider participants spoke about the value that *seeing* each other physically had on the patient-provider relationship. Provider participants felt like they could assess pwCF better if they saw them in-person, which contributed to perceptions of quality clinical encounters:

*“I think anytime you can’t visually see an individual it changes things, … before I worked in CF, I worked in critical care for a long time and so I have a strong sort of understanding … [that] just because somebody looks well or presents well doesn’t mean that that’s actually what’s going on. It’s a lot easier to mask things, right. And the way things were structured [with virtual] it … makes it even harder.”* (provider 5, female)

The importance of in-person, physical assessment was especially valued by pwCF who felt less comfortable verbally describing their condition:

*“I’m not very verbal about my condition with anyone, … [even] my family. Kind of the less-they-know-the-better type thing with me … [because] I don’t want them to get stressed, so I just deal with it on my own. But that can sometimes lead to the appointments … where … I’m used to saying no, I’m good, I’m fine, whatever it is … I can handle it. That is not as easily hidden … if it’s physical … you can’t hide everything, because you don’t look good, you look like you’re sick.”* (patient 8, male)

Patients’ desire to feel *seen* via in-person physical assessment highlights the usefulness of in-person aspects of care to enhance quality during clinical encounters.

*A return to balancing provider-led testing and assessment with patient self-report in shared decision-making.* Pre-pandemic, information used to guide shared decisions between patients and providers appeared to be based on provider-led testing and in-person assessment combined with patient-self-reported accounts. PwCF felt that with shared decision-making, their voices were heard and recognized:

*“They don’t ever force things on me which I really appreciate. They will never say you have to do this … They give me options and say … what’s your opinion, what do you think? Then from there we can make a decision on what we do.”* (patient 8, male)

During the pandemic, there seemed to be a shift in the information used during shared decision-making that stemmed from having to transition from in-person appointments pre-pandemic to mostly virtual (patients could still access the CF clinic during the pandemic if medically necessary but routine management was virtual). Limited access to routine testing and minimal in-person assessments led to an increased reliance on patient self-report:

*“They couldn’t do pulmonary function tests [(PFTs)] during the pandemic. … So, once COVID hit, right away our clinic was closed, unless you were really sick … So … when we’d have the telephone interview, … the doctor … would say, ‘you’ve got to be the one who’s kind of telling us how things are going, because we don’t have a pulmonary function test to determine if there’s been loss of lung function … so you have to tell us how your day-to-day is …[being] impact[ed].’”* (patient 3, female)

Patient and provider participants had mixed feelings on this shift. Provider participants seemed to feel some discomfort and perceived lower quality of care due to the lack of testing and needing to rely heavily on patient self-reported accounts:

*“…there are a lot of visits where we have to fly blind and just go by patient symptoms. So I think for us as healthcare providers it’s a little bit less comfortable because we have less objective data to go by”* (provider 1, female)

In comparison, most pwCF expressed being comfortable with taking more of a leadership role in their care encounters because they had been living with CF their entire lives:

*“[During the pandemic] … I kind of had to make … decisions more on my own. … it was basically my own decision fully during the early throes of the pandemic … I’m pretty self-sufficient when it comes to knowing how severe my condition is or severe my exacerbation is. So, I was pretty comfortable making those decisions”* (patient 2, male)

However, regardless of their level of self-awareness about their condition and how comfortable they seemed to be about taking more of a leadership role, pwCF spoke about wanting pulmonary function testing to validate their self-reports because: “*there was definitely the anxiety of what if we miss something? What if there’s something there that we didn’t really catch? … there’s always a chance that something goes unnoticed*” (patient 8, male).

*Data from self-monitoring could fill the information gap in virtual encounters.* The lack of availability of testing during the pandemic meant that many pwCF did not know the state of their lung function. They also were not being monitored for infections and did not know how long they had an infection if one was eventually found in their system. Not having concrete information about their disease state seemed to cause a sense of worry amongst pwCF:

*“if I’m not there for x-rays …, PFTs … there’s always a chance that something goes unnoticed. … even this last visit that I had yesterday, … I looked decent, I looked healthy, I’m not shedding weight. Everything indicated that I was good, and then the PFT results said otherwise, … So if [I] hadn’t done the PFT, … we might not have caught … a … pretty nasty infection.”* (patient 8, male)

Patient and provider participants offered insights about the utility of home spirometry and how it could have potentially filled the information gap created by a lack of access to testing, consequently decreasing feelings of worry. Some pwCF had purchased a home spirometer at their own expense and considered it useful as they could share their pulmonary function trends with their providers during phone appointments:

*“They didn’t ask what my [exact] readings [were], because it isn’t quite as accurate as a PFT, but they would ask if there was any change. … if I wasn’t feeling great for two or three days in a row, you can just see the numbers going down.”* (patient 3, female)

Provider participants echoed the potential utility of home spirometry and the void it could have filled during virtual appointments. Specifically, provider participants recognized how home spirometry may have allowed them to intervene sooner during the pandemic:

*“I think [home spirometry] … might [have meant we were] … able to intervene sooner if patients had worsening lung [function] – like just thinking about those couple of patients … in the 70s, the 80s, and then two years later we see them when they’re in their 30s, if they were able to do home spiromet[ry] then we would know … [if they] had slowly dropped over time and [they] just never came to see us … … in those cases it would have made a difference.”* (provider 1, female)

Provider participants also recognized how access to home spirometry was not consistent amongst pwCF due to lack of funding and the fact they did not advocate for it as its reliability and validity has not been widely established:

*“It wasn’t accessible to everyone because we didn’t advocate for it. [There was]… some concern about the comparative results between lab and the home device, and [so] we didn’t get [around] to … having a proper consultation among our clinical staff.”* (provider 3, female)

#### 3.2.3 Theme C: Accessibility of appropriate healthcare services could improve efficiency of service delivery.

In theme (c) patient and provider participants spoke about accessibility and appropriateness of multidisciplinary supports and varying modes of delivery, which could subsequently impact efficiency of service delivery.

*Increased availability of appropriate multidisciplinary supports could have allowed for more efficient use of healthcare resources.* Patient and provider participants spoke about the availability of mental health supports, social work, and physiotherapy before and during the pandemic and how each were viewed in terms of appropriateness. Physiotherapy seemed to have inconsistent availability before and during the pandemic: *“[physiotherapy has] been hit and miss over the last several years and so sometimes we may not have that area of expertise readily available for our clinic population”* (provider 4, female). It also seemed to be viewed by pwCF as the least appropriate and unnecessary since they felt they learned necessary skills to manage their breathing challenges as children.

Similarly, social work seemed to be only moderately available in clinic before and during the pandemic. One provider participant identified how the social worker position was funded part-time before and during the pandemic, which is below the staffing standards for the size of the clinic: “*Our current social work FTE is 0.4 which is below the recommended [Canadian staffing] standards for the size of our clinic”* (provider 4, female). The combination of limited social work availability and the need for additional mental health supports as identified by both patient and provider participants illustrates a gap in services. Consequently, provider participants discussed the need to improve access to appropriate mental health supports and social services:

*“We have a social worker that has some availability for counselling, but in terms of in-depth mental health support it’s definitely something we want, but it’s not [something] … we currently have. We did not have [any] prior to the pandemic either. This has actually been an area of keen interest to try and get in the last decade or more for our CF clinic, as with any chronic disease that’s important. And … then add in COVID it’s definitely even more important”* (provider 4, female)

The desire for additional mental health supports to manage aspects of chronic illness was echoed by pwCF, regardless of whether they would access supports themselves:

*“Being able to properly give somebody support when it comes to [mental health in] people with CF … [is important], I think anybody with a chronic illness is going to have some sort of mental illness … So, people that are in the field of chronic illnesses should understand the … actions or steps [that] should be taken when somebody brings up concerns of anxiety or depression or bipolar disorder”* (patient 2, male)

Reallocation of resources devoted to the physiotherapy position to support a mental health practitioner may result in more appropriate care to be available to pwCF when needed, thus ensuring timely and efficient service delivery.

*Flexibility of a hybrid model advances accessibility and convenience of service delivery for patients.* Patient and provider participants discussed positives and negatives of the accessibility, appropriateness, and efficiency of both in-person and virtual service delivery. Both patient and provider participants recognized how virtual delivery was a more efficient use of patient time, making services more accessible for those living rurally, working full-time, or with busy family responsibilities:

*“I like the follow ups over the phone, just convenience wise, it’s 20 minutes out of your day … this morning [at my in-person appointment], I sat there from nine o’clock until 12:30, and so there’s a lot of waiting period in between there. Whereas I could have been doing things at home … And they could phone one at a time and … it’s anywhere between a 5-minute and 20-minute phone call.”* (patient 6, female)

The convenience and flexibility of virtual appointments highlighted in the above quote represents a stark comparison to the amount of time, travel, and costs incurred by out-of-area pwCF attending in-person appointments. Virtual delivery was also highly favoured by pwCF living near the CF clinic as it allowed them to tailor their care to their daily lives. This is essential given that the CF patient population is typically comprised of younger, working-age individuals.

From an appropriateness standpoint, provider participants spoke about how the pandemic broke the “*taboo of telemedicine*” (provider 3, female) by challenging professional boundaries and what was considered acceptable in medical culture:

*“I think at the … professional boundary level, people are starting to feel more comfortable [with virtual] … there w[ere] no other choices whatsoever. … because it wasn’t uniquely … one individual physician, … it didn’t come across that you’re compromising patient care or you’re making up a service which is not culturally acceptable in a healthcare system.”* (provider 3, female)

However, from an efficiency standpoint, provider participants spoke about challenges they faced after switching from in-person to virtual service delivery, including new and additional work tasks:

*“the ones that have phone calls … they still need that spirometry so I was booking a lot more spirometry out of town where they live … there was a lot more of that going on. I’d say that was probably one of the biggest increases in my job, doing that.”* (provider 2, female)

## 4. Discussion

### 4.1 Summary of main findings

We explored patient and provider perspectives of the impact of the COVID-19 pandemic on CF healthcare access and service delivery. Infection prevention at the individual level was not found to be burdensome. Society’s compliance with public health measures, or lack thereof, impacted the level of stigma and anxiety experienced by pwCF. A changed model of care reliant on patient self-report instead of clinician-led testing and in-person assessment due to the transition to virtual care was associated with mixed perceptions since pwCF were comfortable making care decisions but many participants (patient and provider) felt challenged by the lack of objective data for decision-making. It was essential for pwCF to feel known, heard, and seen by their providers in order to feel the care was effective. Finally, critical insights around the need for a balance of in-person and virtual care as well as the need for mental health supports were offered.

### 4.2 Connection to existing literature

Patient and provider participants perceived that pwCF were prepared to deal with the pandemic due to lifelong efforts to prevent infections. This finding is congruent with the published literature. In a qualitative study (n = 17) exploring the concept of social connectedness during the pandemic, the authors noted that pwCF had already been living public health protocols to prevent CF-related exacerbations so COVID-19 protocols were not novel [33]. It was perceived that infection prevention experience created resilience among this population [33]. Resilience and preparedness were also highlighted in a case-control study (n = 4,272) that found that pwCF had lower levels of psychological distress compared to the general public during the pandemic possibly due to the pre-existing experience of pwCF with public health measures [34].

Despite this sense of readiness, there was also increased vigilance by pwCF, providers, and family members/friends to prevent patient exposure to COVID-19. Providers balanced care standards with infection prevention and pwCF balanced increased worry from loved ones with personal frustrations of having to limit activities. Similar to our findings, Harrigan and colleagues (2022) found that some pwCF felt overprotected by loved ones during the pandemic as family members worried about the individual contracting COVID-19 [33]. Among those that did contract COVID-19, our participants perceived outcomes as better than anticipated. Previous research has found that pwCF did not fare worse than the general public [35]. While these studies were conducted earlier in the pandemic, our study was conducted during the rise of the Omicron variant, which suggests these common themes were independent of the dominant COVID-19 variant.

Intriguingly, pwCF in our study discussed how they felt the healthiest they had ever been during the pandemic, which they attributed to the societal adoption of public health measures. This learning is supported by other work. Reviewing data from multiple national CF registries, Burgel and Goss (2021) highlighted how the pandemic resulted in reduced rates of exacerbations amongst pwCF, likely due to social distancing [36]. While the physical effects of society’s adoption of public health measures were positive, patients in our study had mixed emotional experiences. PwCF discussed being faced with both increased stigma and empathy when interacting with members of the public, which are concepts echoed in the literature. Harrigan *et al* (2022) found that some participants felt the pandemic increased societies’ relatability to them and their CF due to increased awareness of respiratory disease and infection control [33]. In comparison, they also heard concerns expressed about how CF-related coughs were stigmatized by the public [33]. Similarly, in a qualitative study (n = 21) exploring experiences of adolescents with CF in Iran during the pandemic, participants discussed how their cough raised fears among those around them as individuals thought they may have COVID-19 [37]. In comparison to our findings, this cough-related stigma was present even among those who knew the adolescents had CF [37], highlighting that the pandemic may have exacerbated the pre-existing stigma associated with chronic illnesses.

Patient and provider participants highlighted the need for increased mental health supports in clinic, during the pandemic and beyond. A study measuring depression and anxiety across 154 CF centres in Europe and the United States found that pwCF and their family members were around two to three times as likely to have elevated symptoms of anxiety or depression when compared to the general community [38]. Poor mental health amongst pwCF has been associated with lower adherence to treatments, worse health outcomes, decreased health-related quality of life, and altered family functioning [39]. Interestingly, a single-centre, comparative study (n = 81) found that mental health of pwCF did not deteriorate during the pandemic but rather showed some signs of improvement [14]. The authors thought these findings may be due to increased contact frequency with the clinic as well as increased support provided to pwCF during the pandemic [14]. However, most CF centers are not properly equipped to manage mental health concerns [39,40] or have the staff to provide a daily touchpoint with pwCF like in the Humaj-Gryszter *et al*. (2022) study. As identified in our study, mental health care typically falls on social workers with already limited time to dedicate to CF clinics [41]. These findings present a gap in current CF care that should be addressed to ensure services are effective at meeting the holistic needs of pwCF. Specifically, we recommend a review of resource allocation in the clinic studied, shifting to prioritize highly valued and desired mental health supports over physiotherapy supports, of which pwCF have more self-perceived comfort with.

We identified that a decrease in access to in-person provider-led testing and assessment during the pandemic resulted in an increased reliance on patient self-reported accounts during shared decision-making. Provider participants discussed feeling discomfort associated with the lack of testing upon which to base their clinical recommendations. Similarly, while pwCF seemed comfortable taking more leadership in care decisions, they worried about exacerbations going unnoticed. The importance of access to testing is echoed by the literature. A survey of telehealth experiences amongst 287 CF programs in the United States during the pandemic found that physical examination and objective testing were necessary for CF decision-making and could not be ascertained properly from telehealth programs [42]. In a study exploring patient and family perceptions of telehealth for CF care (n = 424), those who perceived telehealth as inferior to in-person care identified limited physical assessment and lack of testing as the biggest concerns [41]. This finding is echoed by Womack and colleagues (2020) who highlighted the need for strategies that allowed for collection of necessary testing and laboratory investigations during virtual encounters [43]. The theme of provider discomfort with indirect (virtual) patient assessment combined with patient discomfort with continual self-monitoring indicates that a better balance must be struck between healthcare provider oversight and empowering patients with more ability to perform self-surveillance. This is important in the post-pandemic world and in the age of highly effective modulator therapy where patient needs for healthcare are dropping dramatically and exacerbation symptoms are markedly reduced.

Home spirometry was discussed by patient and provider participants as a means for pwCF to track lung function during virtual appointments. The utility of home spirometry has been demonstrated in the literature though more research into effectiveness and reliability/reproducibility of readings is needed [43] as measurement qualities need to be optimised and access made equitable if implemented [44]. While home spirometry presents a potential adjunct for virtual encounters in the future, a solution for the discrepancy between home and in-clinic devices needs to be established [45]. As such, we support the use of home spirometry to monitor trends in lung function during virtual appointments, or when access to objective testing is limited. However, more research is needed to ensure standardization in measurement.

While in-person care allows for better access to necessary testing and promotes a positive patient-provider relationship, patient and provider participants discussed how virtual care provided flexibility and adaptability to accommodate daily life responsibilities for patients. Similarly, in a qualitative study, Corcoran and colleagues (2023) found that partners of women with CF (n = 20) and their healthcare providers (n = 20) viewed telehealth use during the pandemic as more efficient due to reduced travel time, childcare needs, and in-clinic wait times [17]. PwCF valued the time saved by virtual delivery with some hoping that telehealth would continue in the future [41]. Taken collectively, an optimal approach to future CF care might be a combination of virtual and in-person visits to combine the positive attributes of both.

### 4.3 Policy/clinical practice recommendations

We offer several recommendations for future potential policy changes based on our study findings. First, a model of care with a combination of virtual and in-person care visits should be implemented moving forward rather than resorting back to a strictly in-person delivery model. PwCF felt comfortable leading some care decisions based on their knowledge of their disease and lifetime of monitoring their condition. However, both patient and provider participants discussed the importance of objective data obtained through regular testing, thus necessitating some in-person visits. Second, like most other CF clinics, the clinic recruited from operates under a shared care model where all physicians see all patients. However, the changes in care during the pandemic highlight the benefits of pwCF having one main provider that knows them and their unique healthcare needs. We recommend that each pwCF has one lead physician with others acting as backups to allow flexibility in scheduling, as needed. Finally, participants discussed how multidisciplinary supports varied in availability before and during the pandemic with mental health and social work resources viewed as more appropriate and useful than physiotherapy to pwCF. Since physiotherapy seemed to be inconsistently available, we recommend a reallocation of resources to support a mental health practitioner ensuring more timely access to this resource amongst pwCF.

### 4.4 Limitations

Some limitations may impact transferability of results. First, we interviewed mainly participants who self-identified as female. While we likely could have improved the diversity of pwCF with further recruitment, clinic appointment frequency and feasibility limited availability to do so. Diversity of provider participants was constrained as there is a limited number of providers working in the clinic, so the population to recruit from was small. Second, there was no patient partner on our research team and none of the patient participants wanted to review our findings to determine if they resonated with their experiences. While it would have been ideal to have patient participants review the study findings, the two provider participants felt that they resonated with what they had heard from patients. Third, as this study only recruited from one CF clinic, we cannot not make comparisons about service delivery in different areas. We unfortunately encountered recruitment challenges which prevented us from recruiting participants from more than one CF clinic. This may have limited the diversity of patients and providers interviewed, thus potentially impacting study results. Future research should include larger gender and geographically diverse samples to strengthen transferability and understand if perceptions of access and service delivery differ. Fourth, each study participant was given a $40 electronic gift card to reimburse their time. This amount is not viewed as coercive given the length of the interviews and the fact some participants missed work to participate, but it may have impacted who agreed to participate in the interviews. All the services participants received were free given the publicly-funded nature of Canada’s healthcare system so cost of services did not impact results.

## 5. Conclusions

We studied the impact of the pandemic on CF healthcare access and delivery by integrating patient and provider perspectives to identify implementable strategies for post-pandemic health system improvements. Health system decision makers can use our findings to implement pandemic-related learning to tailor CF services and policies more efficiently and appropriately. Future research efforts should focus on evaluating the effectiveness of any service or system changes made based on our recommendations.

## Supporting information

Appendix 1 CF Paper 071923. Interview guides.
(DOCX)

Appendix 2 Resonance Questions. Questions participants were asked to consider when looking over proposed findings.
(DOCX)

Appendix 3 Exemplary Quotes Table.
(DOCX)

## Abbreviations

CF: cystic fibrosis
pwCF: person with cystic fibrosis
ID: Interpretive Description
SD: standard deviation
PFT: pulmonary function test.
