# Supplementary material for: Living with cystic fibrosis during the COVID-19 pandemic: An interpretive description of healthcare access from patients with cystic fibrosis and their providers in Alberta, Canada
Source: PLoS One. 2025 May 2;20(5):e0322911. doi: 10.1371/journal.pone.0322911 (PMC12047793; doi:10.1371/journal.pone.0322911)
Supplement: Appendix 1 — CF Paper 071923. Interview guides. (DOCX) [file pone.0322911.s001.docx]

Appendix 1 – Interview Guides

**PROVIDER INTERVIEW GUIDE**

INTRODUCTION

Thank you for agreeing to participate in this study. As noted in the informed consent form, your participation is entirely voluntary. You don’t have to answer any questions that you don’t want to. The interview will take about 60-90 minutes. If you would like to take a break for any reason during the interview, please let me know. We can do the interview in multiple sessions, if necessary. I will be recording this interview, if that is okay with you, so that we capture what you say exactly how you say it but all of your contributions will remain anonymous so no one will be able to identify anything that you say. Does that sound okay? Do you have any questions before we begin?

**[START RECORDING]**

1. **We want to better understand the need for, and access to, medical services, like regular clinic visits (both in person and virtual) or emergency department visits for example, by people living with chronic pulmonary disease. To start, I was hoping you could tell me your area of pulmonary care?**

Possible Probes

-How long have you been practicing?

-Have you always worked in this area?

-From your perspective, what unique needs do patients in this area have?

1. **How do you feel your patients’ access to pulmonary care was before the pandemic?**

Possible Probes

-Were there any instances where you wanted to provide medical care but couldn’t due to issues with patient access? What type of medical care and why not?

-Were there any instances where you wanted to arrange access to supplemental care (such as pulmonary rehabilitation or certified respiratory educator or respiratory therapist) but couldn’t?

-Had you experienced any patient barriers to being able to provide care? (be it virtual or in person care)

1. **Could you speak a bit about what you feel your patients’ need for pulmonary care was before the pandemic?**

Possible Probes

-What were the needs with regards to treatment?

-What were the needs with regards to (re)assessments?

-What were the needs with regards to monitoring?

1. **Could you speak a bit about how you feel your patients’ access to pulmonary care may have changed during the pandemic?**

Possible Probes

-Was there a specific experience or reason why you felt like their access to care changed during the pandemic?

-Were there any instances where you wanted to arrange access to adjunctive care (such as pulmonary rehabilitation or certified respiratory educator or respiratory therapist) but couldn’t?

-Did you or your clinic transition to virtual care? Do you feel this impacted patients’ access to care? How?

1. **Could you tell me a bit about whether you feel your patients’ need for medical care may have changed during the pandemic?**

Possible Probes

-What are their current needs with regards to treatment?

-What are their current needs with regards to (re)assessments?

-What are their current needs with regards to monitoring?

6. **Could you speak a bit about the experience(s) that contributed to you feeling like your patients’ need/access for medical care may have changed during the pandemic? => If no changes then can skip to question 7**

Possible Probes

-Was there a difference in when patients sought medical care?

-Was there a difference in the type of medical care being sought? (GP, subspecialty outpatient visit: virtual vs in person, ED)

-Did patients express a preference of in-person versus virtual care?

-Do you have a preference for in-person versus virtual care? Why?

-Did the change in care delivery (in-person vs virtual) impact your ability to provide care?

-How was the contact to your medical care services made? Did it change because of the pandemic?

7. **Could you tell me a bit about how you feel the pandemic has changed your ability to provide care?**

Possible Probes

-Were there interventions or testing (such as Imaging, PFTs, lung rehab) that were limited? Why?

-Was there a time when you chose not to admit a patient that you otherwise would have due to the pandemic?

-Was there a time when you felt you would have rather seen a patient in person and not virtually?

**8. Do you feel as though the medical care you were able to provide adequately addressed your patient’s needs or concerns?**

-To what extent do you feel that your medical care was provided in a safe manner?

-Culturally safe? Respected belief system? Physically safe from an infection perspective?

-To what extent do you feel that the medical care you provided was effective or ineffective as a result of the pandemic?

-To what extent do you feel the medical care you provided was able to meet gold standard of care given the limitations of COVID-19?

1. **What were the costs associated for your patients with the type of management or the method of assessments (ie: virtual vs in-person) you provided over the pandemic?**

Possible Probes

-Financial costs of the service?

-Time? Travel Time? Time off work for themselves and their spouse?

10. **Did you use any self-monitoring tools or devices to help with home assessments for patients?**

Possible Probes

-What types of devices/interventions used?

-If they were not used, do you see the value of using these tools now?

-Have there been any financial barriers to using these remote monitoring tools?

-Have there been any barriers you have perceived with patients accessing these tools?

11.**What barriers, if any, do you perceive might have prevented your patient from trying to access medical pulmonary care?**

12.**With regards to providing pulmonary care during the pandemic, is there anything that stood out as going well?**

Possible probe

- Any novel care delivery systems or components of care that you would keep moving forward?

13.**What do you think could have been done differently to provide pulmonary care during the pandemic?**

Possible Probes

-What could have been improved in the way your patient’s pulmonary needs were cared for or addressed?

-If you could change one experience you’ve had, or didn’t have, in providing pulmonary care during the pandemic, what would it be? Why?

14.**Do you think the pandemic has influenced (positively or negatively) the pulmonary health outcomes of your patients? Why or why not?**

Possible Probes

-Did many patients acquire COVID-19 infection that you are aware of?

-Did the acquisition of COVID-19 infection adversely impact the health outcomes of your patients?

15.**Looking forward, what are your expectations for providing pulmonary care for the rest of the pandemic? What about after the pandemic?**

16.**Is there anything else that we have not discussed but that you would like to share?**

**[STOP RECORDING]**

Finally, can you share basic demographic information for analysis purposes, including your age, gender, years in practice and ethnicity?

**PATIENT INTERVIEW GUIDE**

INTRODUCTION

Thank you for agreeing to participate in this study. As noted in the informed consent form, your participation is entirely voluntary. You don’t have to answer any questions that you don’t want to. The interview will take about 60-90 minutes. If you would like to take a break for any reason during the interview, please let me know. We can do the interview in multiple sessions, if necessary. I will be recording this interview, if that is okay with you, so that we capture what you say exactly how you say it but all of your contributions will remain anonymous so no one will be able to identify anything that you say. Does that sound okay? Do you have any questions before we begin?

**[START RECORDING]**

1. **We want to better understand the need for, and access to, medical services, like regular clinic visits (both in person and telephone/virtual/telemedicine) or emergency department visits for example, by people living with chronic pulmonary disease (such as asthma, COPD, cystic fibrosis, pulmonary hypertension) during the ongoing COVID-19 pandemic. To start, I was hoping you could tell me your [pulmonary condition] story?**

Possible Probes

-When did you first start noticing your symptoms?

-When did you get diagnosed?

-How has living with [pulmonary condition] affected your life?

-How do you feel that your symptoms have interfered with your daily home and work activities? What about your social relationships?

1. **Could you tell me a bit about what your need for pulmonary medical care was like before the pandemic?**

Possible Probes

-What were your needs with regards to treatment?

-What were your needs with regards to (re)assessments?

-What were your needs with regards to monitoring?

1. **Could you tell me a bit about your level of access to medical care before the pandemic?**

Possible Probes

-Were there any instances before the pandemic where you wanted to access medical care or testing but couldn’t? What type of medical care and why not?

1. **Could you tell me a bit about whether you felt like your need for pulmonary care changed during the pandemic?**

Possible Probes

-How did your needs change with regards to treatment of disease/symptom flares? Was this identified by the doctor or by you?

-How did your needs change with regards to (re)assessments of disease/symptom flares? Was this identified by the doctor or by you?

-How did your needs change with regards to monitoring of disease/symptom flares? Was this identified by the doctor or by you?

-Was there a specific experience or reason why you felt like your need for medical care changed during the pandemic?

1. **Could you tell me a bit about whether you feel your access to medical care changed during the pandemic?**

Possible Probes

-Was there a specific experience or reason why you felt like your access to medical care or testing changed during the pandemic?

-Were there any services or tests that you wanted to access during the pandemic but couldn’t? Why not?

6. **Could you walk me through what your ongoing pulmonary medical care is like?**

Possible Probes

-What health care providers have you accessed during the pandemic?

-What have they done/what interventions did you receive?

-When did you seek medical care?

-What led you to seek medical care?

-Was there someone that encouraged you to seek medical care?

-Did you seek medical care on your own or were you guided by a healthcare professional?

-Were there any particular symptoms that you were experiencing that made you more motivated to seek medical care initially?

-How was the contact with the medical care services made?

-How did you feel about the process?

-Could you speak a bit about whether you’ve received any psychological or social work support or accommodations to allow access to medical care?

**7. Only if you feel comfortable disclosing, did you acquire COVID-19 infection?**

- If so, how did this impact your pulmonary care needs and access?

8. **Could you tell me a bit more about your thoughts on the quality and effectiveness of these experiences with your pulmonary health care provider(s), before and during the pandemic?**

Possible Probes

-How would you describe your medical providers’ approach with you?

-Person-centered? Do you feel like the medical providers you encountered understood your needs?

-Do you feel as though the medical care you received addressed your needs or concerns?

-To what extent do you feel that you got the answers or information you needed?

-To what extent do you feel that your medical care was provided in a safe manner?

-Culturally safe? Respected belief system? Physically safe from an infection perspective?

-To what extent do you feel that the medical care you received was effective?

-Did it match the symptoms you were experiencing?

9. **Did you receive virtual care (telephone or video) during the pandemic and if so, do you feel this changed your quality of care compared to in-person care? => If no change, then skip to 10**

Possible probes

-(If virtual care negatively affected care): In what way do you feel virtual care resulted in poorer quality of care?

- Do you feel your health outcomes were negatively impacted by this?

-(If virtual care positively affected care): In what way do you feel virtual care resulted in improved quality of care?

- Do you feel your health outcomes were improved by this?

-Did you receive telephone care, video care or both? Do you have preference for one over the other?

1. **What were the costs associated with the treatments you received?**

Possible Probes

-Financial costs of the service?

-Time? Travel Time? Time off work for themselves and their spouse?

11.**To what extent have you managed your symptoms on your own during the pandemic?**

Possible Probes

-Did you choose to manage your symptoms on your own?

-Would you have liked to have more support? What support do you feel you needed?

12. **Did you use any self-monitoring tools or devices (such as home spirometers, Fitbits/Apple watches, oxygen probes, blood pressure cuff or weight scales) to help with home assessments?**

Possible Probes

-What types of devices or interventions did you use?

-Did you experience any financial barriers limiting your ability to use these remote monitoring tools?

-Did you have to buy them yourself or did the clinic provide them?

-Were there any other barriers (other than financial) that you might have experienced when accessing these tools?

13.**What barriers, if any, prevented you from trying to access medical pulmonary care during the pandemic?**

Possible Probes

-Was there a time(s) when you did not seek medical attention when you otherwise would have? What were the reasons for this?

-What about getting lab, imaging or other diagnostic tests?

14.**In your pulmonary care journey during the pandemic, is there anything that stood out as going well?**

15.**What do you think could have been done differently along your pulmonary journey during the pandemic?**

Possible Probes

-What could have been improved in the way your pulmonary needs were cared for or addressed?

-If you could change one experience you’ve had, or didn’t have, during your pulmonary journey during the pandemic for the next person to make their journey better, what would it be? -Why?

16.**Looking forward, what are your expectations for your pulmonary care for the rest of the pandemic? What about after the pandemic?**

17.**Is there anything else that we have not discussed but that you would like to share?**

**[STOP RECORDING]**

Finally, can you share basic demographic information for analysis purposes, including your age, gender, living in urban/rural setting and ethnicity?
