# Supplementary material for: Living with cystic fibrosis during the COVID-19 pandemic: An interpretive description of healthcare access from patients with cystic fibrosis and their providers in Alberta, Canada
Source: PLoS One. 2025 May 2;20(5):e0322911. doi: 10.1371/journal.pone.0322911 (PMC12047793; doi:10.1371/journal.pone.0322911)
Supplement: Appendix 2 — Resonance Questions. Questions participants were asked to consider when looking over proposed findings. (DOCX) [file pone.0322911.s002.docx]

Appendix 2 – Questions participants were asked to consider when looking over proposed findings.

1. Do these findings resonate with your experience of providing CF care during the pandemic?
2. If an idea does not resonate, why not? What could we change to make it resonate more?
3. Is there anything key to your experience that you think is missing?
