# Supplementary material for: Living with cystic fibrosis during the COVID-19 pandemic: An interpretive description of healthcare access from patients with cystic fibrosis and their providers in Alberta, Canada
Source: PLoS One. 2025 May 2;20(5):e0322911. doi: 10.1371/journal.pone.0322911 (PMC12047793; doi:10.1371/journal.pone.0322911)
Supplement: Appendix 3 — Exemplary Quotes Table. (DOCX) [file pone.0322911.s003.docx]

Appendix 3 – Exemplary quotes table

| **Main Theme** | **Subthemes** | **Exemplary Quotes** |
| --- | --- | --- |
| Tensions due to pandemic-related infection prevention at micro-, meso-, and macro- levels |  |  |
|  | Micro: PwCF felt prepared yet vulnerable | “…obviously, no one wants to get COVID … but I think for somebody who has CF, … I wouldn’t say they’re de-sensitised to it, but every year we kind of deal with this – we don’t want to get sick, because as somebody has a CF, if you get sick you can end up hospitalised … from just a common cold or a flu. So for me this didn’t feel like anything new.” (male patient, 019)  “There’s always flus and colds going around, so … the current procedures [are] … pretty much what they were doing pre-COVID with all the CF patients. They're kind of [were] treating us all like little COVID patients, pre-COVID.” (female patient, 011)  “Living with cystic fibrosis [has]… played into my career a little bit … part of that reason is so that I’m not on the medical unit … But that was actually a discussion that I had with my healthcare team … And so that’s how I ended up in [the field I work in].” (female patient, 014)  “I would kind of always talk with the patients and I would kind of always, you know, comment that really in the context of the pandemic they’ve been training for this their whole life. They understand the importance of like staying healthy and washing their hands and mitigating risk” (female provider, 012)  “So for our patients it’s kind of like they were living COVID protocol … their entire lives. And then it’s just the rest of the world caught on” (female provider, 001) |
|  | Meso: Balancing act between infection prevention and access to care | “But [the clinic] definitely had their hands full, like it’s people who were immunocompromised that they’re dealing with constantly, right, so it’s very taxing for them as well, not just the patients, but just for the staff involved. They have so many basically sick patients that need to come in, but again it’s a pandemic, so it’s they’re playing a game of do we want these patients in right now and then there’s the risk of if one of them uses a PFT machine, you know that there’s a – if they can’t really clean that out inside out the way they want to, and if that could spread to another patient. So they had to be very, very careful of that.” (male patient, 019)  “…especially at the beginning of the pandemic in 2020, we opted to not have bloodwork done to minimize … number one, it was difficult to get into the lab. Number two, we weren’t sure if they would – like people were uncomfortable going into the lab and potentially being exposed to others to get the bloodwork drawn.” (female provider, 008)  “… there was an expectation setting kind of thing because we were worried that our CF patients would be more vulnerable to COVID and that they would be worse if they COVID but we actually didn’t see that. So it wasn’t like they were more susceptible and when they did get COVID it wasn’t like everybody went to the ICU. So yes, because the early days it was really bad that they – there was like the national sort of ICU triaging protocol and basically any CF patients with lung function less than 40 percent you were not going to go to ICU. So yes, so we were all prepared for the worst” (female provider, 001)  “I think the care during the pandemic was maybe more informative and the quality was probably a little bit better, because maybe they were on high alert, just because everybody would see us as more on the more riskier zone, right. So, the quality was definitely maybe even more than prior to the pandemic” (female patient, 015)  “in the beginning of the pandemic, I mean, I'm sure almost anybody with any kind of a comorbidity was a little bit anxious, because it’s respiratory, right.” (female patient, 009)  “I also used to do a lot of hot yoga. And so just being really aware that you’re in a very small room – you know, next to you a lot of people working out and breathing in and out and all that stuff. So I haven’t actually been to yoga since the pandemic started.” (female patient, 014) |
|  | Macro: Society’s adoption (or non-adoption) of public health protocols created physical and emotional tensions | “People … maybe understand a bit more how difficult it might be to live with CF. Because maybe people are thinking more about the difficulty of having a chronic lung condition because [COVID] is based on the lungs … But then also just … feeling maybe stigma surrounding having a chronic cough. And not wanting people to worry that … I have COVID or something, when this is how I’ve been coughing my whole life.” (male patient, 005)  “I’m thinking about a time when I was in [a grocery store]. I was standing in line and I was just at the front of the line and there’s this older gentleman behind me, and I had a coughing fit, because my lungs are mucousy obviously due to the CF, but he doesn’t know that. And I started coughing like crazy – I had my mask on and everything’s good, I’m covered and everything. He turned and looked at me and he actually left the line and went to a different till that had a line that was like 10 times longer, because he just didn’t want to be around me … I think that was definitely increased during COVID, I get funny looks and it is what it is, right. There’s anxiety attached to this.” (male patient, 019)  “I would say [stigma] was increased … because it really came to a point where people were just like so hyper-vigilant, right, and you would hear a cough and you would think like ‘oh my goodness, like why is this person out in the community.’ And it just became – at least from what I was seeing in a lot of places like it was just automatically associated with COVID.” (female provider, 012)  “And so, you can just, like being in proximity to a maskless person can really raise my anxieties around me going out in public and stuff. Because I was aware that a lot of people were refusing to comply. So, just it kind of forced me to hermit more than I’d like to, I would, still wanted to go and do shopping and be able to just go out on walks or whatever. But just being concerned that I might cross paths with somebody who was not compliant with the guidelines stopped me from pursuing activities outside of my house a lot of the time.” (male patient, 005)  “And we’ve certainly had patients … tell us … that like, yes, the last two years I felt the best I’ve ever felt because I wasn’t getting sick all the time and my children were at home so they weren’t bringing home things and so I wasn’t getting sick every month. So for all those reasons we definitely have heard people say … exactly those words, they’ve felt the best that they felt because [of] the public health measures” (female provider, 001)  “[everyone else] wearing the mask really did make a difference … I felt my healthiest I’ve ever been” (female patient, 017) |
| Modifying aspects of person-focused care can bolster perceived quality of clinical encounters |  |  |
|  | Feeling known, heard, and seen lays the foundation for meaningful patient-provider relationships | “They don’t ever force things on me which I really appreciate. They will never say you have to do this, and you’re going to do this and you’re going to this. They give me options and say what do you think is the best – like what’s your opinion, what do you think? Then from there we can make a decision on what we do. So yeah, that’s always appreciated.” (male patient, 019)  “A few years back I had gotten quite sick – and this was pre-pandemic – … and I went into clinic, and I knew I needed an IV. But the doctor said to me, ‘I think we need to do IV antibiotics; how do you feel about this?’ And I was like, ‘That’s why I'm here.’” (female patient, 009)  “it’s usually how it goes in appointments, that they kind of trust that you know your body better than they do, to a certain degree. So, if you are experiencing things, then usually they … will [trust your perspective]” (female patient, 011)  “[During the pandemic] I would take more into account my own interpretation of what my condition was … I think there was one time where I had to decide whether I was going to go on IV medication or just oral medication. So, I kind of had to make those decisions more on my own. They do take into account my opinion when it comes to when I'm doing IV or oral antibiotics to deal with an exacerbation, but it was basically my own decision fully during the early throes of the pandemic.” (male patient, 005)  “They couldn’t do pulmonary function tests during the pandemic. … So, once COVID hit, right away our clinic was closed, unless you were really sick, and then you could go into clinic. But everything was a telephone interview. So you know, when we’d have the telephone interview, … the doctor … would say, ‘you’ve got to be the one who’s kind of telling us how things are going, because we don’t have a pulmonary function test to determine if there’s been loss of lung function, that kind of thing, so you have to tell us how your day-to-day is …[being] impact[ed].’” (female patient, 009)  “we have – I think it’s four doctors. … I went for my yearly clinic, and … The doctor … called me by the wrong name. I don’t think [they] had the right chart up on the computer. … so from that point forward, I always see the same doctor, and I’ve made it quite clear, unless it’s an emergency, I only want to see the one doctor … Sometimes you can just do a visual assessment on people to see if it’s good, bad, whatever. So, once I switched to a single doctor – and that would have been a couple of years ago – my quality of care has been – I have nothing but good things to say about it.” (female patient, 009)  “… our patients know that they can just call the clinic. They can speak with a nurse, they can speak with the NP, so there’s somebody there all the time that they know and that they can talk to. So that’s kind of the unique thing about [CF], whereas other lung conditions like they don’t expect that, they wouldn’t have that capability” (female provider, 001) |
|  | A return to balancing provider-led testing and assessment with patient self-report in shared decision-making | “I feel like [the pandemic] has [changed my ability to provide care]. Again, I think maybe the outcomes don’t show that and maybe we’ve always just been too conservative as a practitioner group where we can see patients so frequently. But I certainly feel a little bit of a trepidation myself that, you know, it’s not how I trained. We all trained to see our patients every three months then we would have pulmonary function testing and we’d be able to examine them and things like that.” (female provider, 001)  “There was definitely the anxiety of what if we miss something? What if there’s something that there that we didn’t really catch? Like for me it was way more convenient to do it over the phone, because I don’t have to drive there, I don’t have to go to the office, I don’t have to go downstairs to do bloodwork or anything like that, it’s just over the phone and it’s simple and it’s done. But there was always the anxiety of what if we missed something because they can’t take my vitals. They can’t – you know if I’m not there for x-rays or whatever they need me to do, PFTs and that stuff, there’s always a chance that something goes unnoticed” (male patient, 019)  “In-person would probably be – I mean, the phone call was great ‘cause I didn’t have to drive the distance, but I do prefer to go in because … I used to know my health so well that I could tell you exactly if it’s a cold, if it’s an exacerbation, you name it, I can be like oh, this is what’s going on. Since I started Trikafta I – it’s completely flipped everything upside down so I was having exacerbation and I thought it was just like, the sniffles and I was like oh it’s fine. But then my lung function actually dropped like five percent, without me really knowing so the fact that I was able to go in showed that and they kind of caught it in time whereas if I would have just been like oh it’s nothing, and then just stay on a phone call well we wouldn’t have caught it” (female patient, 017)  “I’m not very verbal about my condition with anyone – I’m not [laughs] very honest about it with my family. Kind of the less-they-know-the-better type thing with me. I know it’s not fair for me to do that to them obviously, but … I don’t want them to get stressed, so I just deal with it on my own. But that can sometimes lead to the appointments, right, where I’ll – I’m used to saying no, I’m good, I’m fine, whatever it is I can deal with it – I can handle it. That is not as easily hidden, because there’s a physical aspect involved if it’s physical– you look like shit right now, but you don’t – you can’t hide everything, because you don’t look good, you look like you’re sick. So it definitely would be, I think, beneficial in the just visual aspect involved.” (male patient, 019)  “I think in‑person care is still more thorough, less chance of missing things. I try my best to see new patients in‑person because I don’t feel that I have as good of an assessment, a first assessment for patients that I’ve only met by Zoom or by phone. Yeah, but definitely I think that there is a role for it.” (female provider, 008)  “I think [virtual care] does [impact outcomes]. I think anytime you can’t visually see an individual it changes things, right, because it takes away like the element – I don’t know, before I worked in CF, I worked in critical care for a long time and so I have a strong sort of understanding of like just because somebody looks well or presents well doesn’t mean that that’s actually what’s going on. It’s a lot easier to mask things, right. And the way things were structured in just trying to like get individuals to like follow-up, call back, those kinds of things it even makes it even harder because you might have only maybe like little, tiny snippets of time where you might not be able to slowly coax them into a conversation and really do a deep dive.” (female provider, 012) |
|  | Data from self-monitoring could fill the information gap in virtual encounters | “They didn’t ask what my readings are, because it isn’t quite as accurate as a PFT, but they would ask if there was any change. And I mean, it can change from hour to hour, you know, it’s just a little window. … If I wasn’t feeling well, I'd generally do it three or four times a day, then I have a bigger picture of … where it was at. But it would also tell me, if I wasn’t feeling great for two or three days in a row, you can just see the numbers going down.” (female patient, 009)  I think [home spirometry] … might [have meant we were] … able to intervene sooner if patients had worsening lung – like just thinking about those couple of patients where it started off like in the 70s, the 80s, and the two years later we see them when they’re in their 30s, if they were able to do home spirometer then we would know like right now we’re seeing you again in your 30s like is this an acute prob[lem] or is this something that you had slowly dropped over time and you just never came to see us and the results of significant exacerbation or, you know? So yes, I think definitely in those cases it would have made a difference.” (female provider, 001)  “… we could have been a bit more proactive, because it depends how you approach things … you can be more pro something and the people, they do trust us, you know what I mean. So if we say this works, this may minimise the number of times you come to clinic, but the caveat is it’s not going to be identical same as you’re PFT lab results, but you’re going to use this device as tool of how things [are] with you and your symptoms at home, and then flag it up to us. … the trend matters. I don’t think retrospectively if we knew we were going to be hit by the pandemic, then probably we would equip like majority of our patients with this home device. Because it could have helped us a lot of time when we were scratching our head and then we’re debating is she, or is he sick, do we need to see them – do you know what I mean?” (female provider, 007) |
| Accessibility of appropriate healthcare services could improve efficiency of service delivery |  |  |
|  | Increased availability of multidisciplinary supports could have allowed for more efficient use of healthcare resources | “You know what physio was hit or miss, social worker was hit or miss as well.” (male patient, 019)  “In terms of the fact that we are a multi‑disciplinary clinic and our patients have multi‑disciplinary needs I would say that the care that we provide may be limited by our staffing supports and we may not have – we don’t have a dedicated pharmacist in our clinic so we would try and enlist the help of a general pharmacist, for instance, or we haven’t always had access to a physiotherapist. It’s been hit and miss over the last several years and so sometimes we may not have that area of expertise readily available for our clinic population.” (female provider, 008)  “For me, personally, … for the most part I know what I need to do kind of stuff … I kind of already know what to do for the exercising route [for airway clearance], it’s just a matter of me actually doing it, I guess. (female patient, 017)  “We have a social worker that has some availability for counselling, but in terms of in‑depth mental health support it’s definitely something we want, but it’s not a resource that we currently have. We did not have one prior to the pandemic either. This has actually been an area of keen interest to try and get in the last decade or more for our CF clinic, as with any chronic disease that’s important. And then chronic disease – and then add in COVID it’s definitely even more important, yeah. Unfortunately, we don’t. Our current social work FTE is 0.4 which is below the recommended standards for the size of our clinic, for the Canadian standards for staffing.” (female provider, 008)  “Being able to properly give somebody support when it comes to [mental health in] people with CF … or anybody with a chronic illness, I think anybody with a chronic illness is going to have some sort of mental illness it seems as well, it's a very big correlation. So, people that are in the field of chronic illnesses should understand the, maybe what actions or steps should be taken when somebody brings up concerns of anxiety or depression or bipolar disorder, whatever it is. Just they're very highly correlated and related like chronic illness and mental illness” (male patient, 005)  “My only thing I think I could say about the CF clinic is with … mental health they should have the therapists and the counsellors and things like that for people. I don't know if they actually do or not and I actually don't know if I've ever asked, but it's never ever really been offered to me. That is something I would have had to have done on my own, right. So, I just think when you come with a chronic condition, you're going to come with some backlash with it” (female patient, 015) |
|  | Flexibility of a hybrid model advances accessibility and convenience of service delivery for patients | “So they went to more virtual. They weren’t pushing for in-person, which is fabulous because I live [rurally]. And then either a stayover night, depending if I have childcare or my husband’s home or whatever, or I just do it all in one day” (female patient, 005)  “I like the follow ups over the phone, just convenience wise, it's 20 minutes out of your day and even if you have to talk to a couple of people, it’s whenever they get the chance they can phone you, you can still. I, this morning, I sat there from nine o'clock until 12:30, and so there's a lot of waiting period in between there. Whereas I could have been doing things at home or whatever I needed to do. And they could phone one at a time and as long as I have the time and it’s anywhere between a five minute and 20-minute phone call, so it's kind of nice to have the phone follow ups.” (female patient, 015)  “I think at the kind of clinical level, like a professional boundary level, people are starting to feel more comfortable. Because it wasn’t one or two people … that offered telephone visits. Like it wasn’t like oh OK, I am compromising patient care, do you know what I mean – so there was no other choices whatsoever. So with the available resources, we still wanted to be available for our patients, then we didn’t have any other choices than at least talking to them over the phone and get the sense that – or triaging them at least over the phone. And then because it wasn’t uniquely for one individual physician, so it didn’t come across that you compromising patient care or you’re making up a service which is not culturally acceptable in a healthcare system” (female provider, 007)  “…you had to sit down and come up with ideas on how are we going remotely manage our patients. And then that … broke the taboo of telephone visit …, [the idea that] I have to see you” (female provider, 007)  “I think we all did a pretty good job with what we were presented with. I think one of the things that has happened is that it’s kind of nudged health care into providing alternative ways of care that support those more rural patients or, you know, ones who have barriers to coming into health care. But I think considering the rapid way that things changed and how things just shut down so quickly, I think like we’ve done a pretty good job.” (female provider, 012)  “Our physicians work very closely with our medical office assistants and when the medical office assistants were working virtually it became very difficult to do things which sometimes are much easier done in‑person. For instance, if I needed to fax forms in general I would have my medical office assistant helping out with that. However, if they were virtual then I would have to do my own faxing, my own scanning, that sort of thing. Of course it takes away from patient care because I’m spending the time doing a role that somebody else is there to do, but just not physically present and able to help” (female provider, 008) |
